# Electrical Tree Characteristics of Bisphenol A Epoxy Resin/Maleopimaric Anhydride Curing System

**DOI:** 10.3390/polym14183867

**Published:** 2022-09-15

**Authors:** Hechen Liu, Xuan Wu, Zhanpeng Guo, Peng Dong, Qi Ge, Liwei Wei, Zhanglin Sun

**Affiliations:** 1 State Key Laboratory of Alternate Electrical Power System with Renewable Energy Sources, North China Electric Power University, Yonghua North Street No. 619, Baoding 071003, China; 2 Hebei Key Laboratory of Green and Efficient New Electrical Materials and Equipment, North China Electric Power University, Yonghua North Street No. 619, Baoding 071003, China; 3 State Grid Chongqing Electric Power Company, Qingfeng North Road No. 20, Chongqing 401121, China

**Keywords:** bisphenol A epoxy resin, maleopimaric anhydride, electrical tree, molecular dynamics

## Abstract

Epoxy resin insulation materials are mainly derived from petrochemical materials which have the disadvantages of resource consumption and environmental pollution. In order to cure bisphenol A epoxy resin, a maleopimaric anhydride (MPA) curing agent was prepared from rosin, a renewable resource, and blended with a petroleum-based curing agent (methylhexahy-drophthalic anhydride). The influence of maleopimaric anhydride content on the initiation and growth characteristics of electrical trees was studied and analyzed in this paper using molecular dynamics simulation (MD) and electrical tree tests at an 18-kilovolt power frequency voltage. When the MPA content used was ≤10%, the free volume percentage of the curing system increased with MPA content, and thus the initiation voltage became lower; when the MPA content was ≥20%, the hydrogenated phenanthrene ring structure content increased significantly with increasing MPA content, and the rigidity of the curing system increased significantly; thus, the initiation voltage gradually increased. MPA4 had an 11.11% higher initiation voltage than the petroleum-based control group. The effect of the polar rigid structure within the curing system significantly inhibited the growth rate and length of electrical trees as MPA content increased. Electrical trees developed into light-colored, thin, and narrow dendritic structures when the MPA content reached 40%. The results show that curing epoxy resin with the rosin-based curing agent maleopimaric anhydride (MPA), in place of a petroleum-based curing agent, can produce environmentally friendly resins with excellent electrical tree resistance and potential application prospects.

## 1. Introduction

In recent years, over-exploitation of natural resources has resulted in an imbalance between the supply and demand for petrochemical resources, and also an increase in environmental pollution [1,2]. Modern industry’s over-reliance on petrochemical raw materials has resulted in a sharp decrease in oil reserves, an imbalance in the carbon cycle, and excessive sulfur gas standards [3,4]. With growing awareness in the importance of resource conservation and environmental protection, the search for non-polluting and renewable materials to replace petrochemicals has become an unavoidable trend for sustainable long-term development. As a result, replacing petroleum-based electrical equipment with biomass materials has become a focus of research in the field of electrical materials. Extensive research has been conducted on vegetable oil transformers (using vegetable oil to replace mineral oil), environmentally friendly GIS insulating gas, recyclable power cables, and so on [4,5,6,7,8]. Some of the research findings have been put into production and use, resulting in significant environmental and economic benefits.

Petroleum-based bisphenol A epoxy resin (DGEBA) is widely used in the insulating castings of electrical equipment, such as in dry-type transformers. It has the advantages of being a good electrical insulator, possessing strong weather resistance, having excellent mechanical properties, and involving a simple, cost-effective process [9,10]. However, in order to reduce the excessive dependence on petroleum-based raw materials, the use of natural renewable resources to replace petroleum-based raw materials for the synthesis of environmentally friendly epoxy resins with excellent performance has become a hot research topic [6,11].

**Figure 1:**
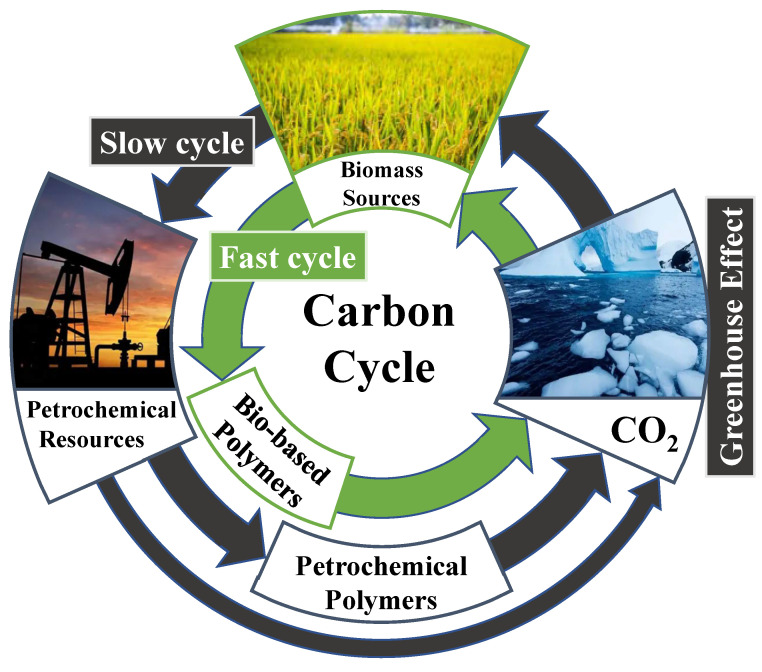
Carbon cycle diagram.

It takes millions of years to form non-renewable petrochemical resources from renewable biomass resources in nature [7]. Therefore, using biological resources to directly synthesize polymer materials that replace petroleum-based polymer materials can not only solve the resource problem, but also accelerate the process of the carbon cycle in nature and thereby reduce the content of greenhouse gases in the atmosphere [12]; such an approach can be beneficial for ecological protection, as shown in Figure 1.

In existing studies, natural renewable biomass resources, such as starch, green tea, lignin, rapeseed oil, itaconic acid, rosin, etc., have been used to synthesize bio-based epoxy resins and bio-based curing agents [11,13]. However, at present, bio-based epoxy resins have not yet met the application requirements of electrical equipment, in terms of insulation performance. Among them, epoxy resin prepared by rosin has excellent properties of high-temperature resistance, corrosion resistance, and UV aging resistance; furthermore, rosin contains a stable hydrogenated phenanthrene ring rigid structure and active functional groups, such as double bonds, that can be easily chemically modified. Therefore, rosin can be used to prepare the bio-based curing agent maleopimaric anhydride, after which the epoxy resin is cured in order to introduce the hydrogenated phenanthrene ring rigid structure into it [14].

Previous research from our group showed that epoxy resin cured by a petroleum-based curing agent partially replaced by MPA demonstrates excellent performance in insulation field strength tests, dielectric property tests, and leakage current tests. This tentatively proves that rosin-based epoxy resin has excellent insulation performance, and has the prospect of being applied in the field of electrical materials. Therefore, this paper further investigates the electrical tree resistance of rosin-based epoxy resins and explains the phenomenon at the microstructure level.

During the production and use of polymer insulating materials, their electrical characteristics undergo irreversible changes under the long-term effects of electricity, heat, mechanical force, light, oxygen, and radiation [15]. When there are local cracks, particle impurities, or bubbles in the insulating material, it is easy to distort the local electric field, which in turn causes partial discharge and the formation of electrical trees [16]. The phenomenon of electrical dendritic degradation is a comprehensive process that includes charge aggregation and migration, localized field formation, mechanical stress, chemical decomposition, electroluminescence, and localized high temperature. It is widely believed that the growth of electrical dendrites is closely related to carrier migration behavior in insulators and their localized field formation; moreover, the breakage of polymer molecular chains and the formation of free radicals are the hallmarks of electrical dendrite initiation.

Under AC voltage, the low voltage, fast growth rate, and destructive nature of electrical trees are the main factors triggering insulation breakdown in the long-term operation of insulating materials [17]. As the transmission capacity of the power grid continues to increase, the voltage level continues to increase, and the external environment becomes more complex; the problem of aging of the electrical trees in the insulation material becomes increasingly prominent. Therefore, the electrical tree resistance of insulating materials can be used as an important basis for assessing their insulation performance. At present, methods such as improving the manufacturing process and material properties are usually used to suppress the initiation and growth of electrical trees [18].

In this paper, epoxy resins were modified using bio-based curing agents as a partial replacement for petroleum-based curing agents. The bio-based curing agent maleopimaric anhydride (MPA) was synthesized by using the natural material rosin, and the petroleum-based curing agent methyl-hexahydro phthalic anhydride was partially replaced by MPA in order to cure the epoxy resin DGEBA. The effect of MPA on the electrical tree resistance of the blended system was tested and analyzed using the electrical tree test and molecular simulation, in order to finally determine the optimal ratio to improve the insulation performance of the blended system.

## 2. Materials and Methods

### 2.1. Materials

The materials used in this paper included bisphenol A epoxy resin (E-51, analytical industrial purity, 0.45–0.51, Shanghai Resin Factory, Shanghai, China); maleopimaric anhydride (MPA, laboratory-made); methyl hexahydrophthalic anhydride (MHHPA, 98% purity, Zhejiang Qiyi Electric Co., Quzhou, China); and accelerator DMP-30 (96% purity, Zhejiang Qiyi Electric Co., Quzhou, China).

Preparation of MPA: A mass of 50 g of rosin was added to a 1000-milliliter three-necked flask that was equipped with a condenser tube, a magnetic stirrer, a thermometer, and a nitrogen gas conduit. Then, the temperature was raised in an oil bath under nitrogen protection and maintained at 140 °C for 3 h, in order to isomerize rosin to levopimaric acid. The system was then slowly cooled to 110 °C, after which 30 mL of glacial acetic acid, 11 g of maleic anhydride, and a certain amount of p-toluenesulfonic acid as catalyst were added, and the system was reacted under reflux for 3–5 h; then, 100 mL of glacial acetic acid was added when the material temperature dropped to 90 °C, followed by mixing evenly. When the material cooled down to room temperature, it was further cooled with 0 °C deionized water, and filtered to obtain MPA as a white crystalline crude product. It was then recrystallized with glacial acetic acid 2–3 times, and dried under vacuum to obtain MPA.

### 2.2. Preparations

The theoretical mass ratio of epoxy resin and curing agent was calculated according to the epoxy equivalent. MPA and MHHPA were blended proportionally, where the blending ratio K = amount of anhydride in MPA/amount of total anhydride in the curing agent.

**Figure 2:**
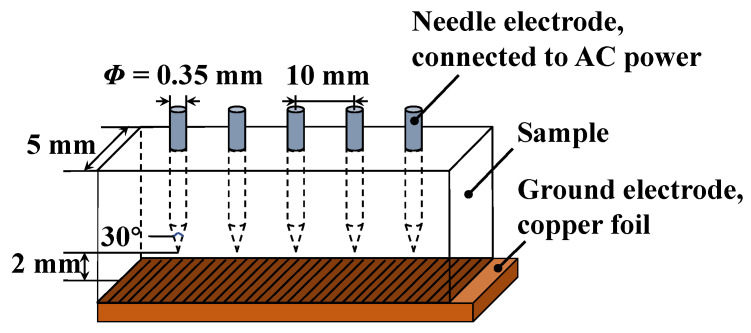
Electrical tree sample model.

Firstly, the two curing agents MPA and MHHPA were mixed according to the five proportions listed in 0, and then co-mixed with the equivalent amount of bisphenol A type epoxy resin (DGEBA). This was then stirred for 15 min under the condition of 45 °C in a water bath until well mixed. After that, 0.5 wt% of accelerator DMP-30 (based on the total mass of curing agent and resin) was added, and the mixture was stirred for 10 min at 60 °C in a water bath until it became homogeneous. After that, the mixture was vacuum defoamed for 30 min at an ambient temperature of 60 °C. Finally, using the needle electrode pre-embedded method, the mixture after vacuum debubbling was injected into the mold treated with a release agent and preheated (60 °C). It was then placed into a vacuum drying oven for high-temperature curing, under conditions of 100 °C/2 h, 120 °C/2 h, and 150 °C/5 h. After removing the cured specimen, the conductive copper foil was adhered reliably at the bottom of the specimen with a strong adhesive to form the needle-plate electrode structure. The structures of the specimen and needle electrode dimensions are shown in Figure 2, with a needle electrode diameter of 0.35 mm, a radius of curvature of 3 μm, and a vertical distance of 2 ± 0.05 mm from the tip of the needle electrode to the plate electrode.

### 2.3. Methods

**Figure 3:**
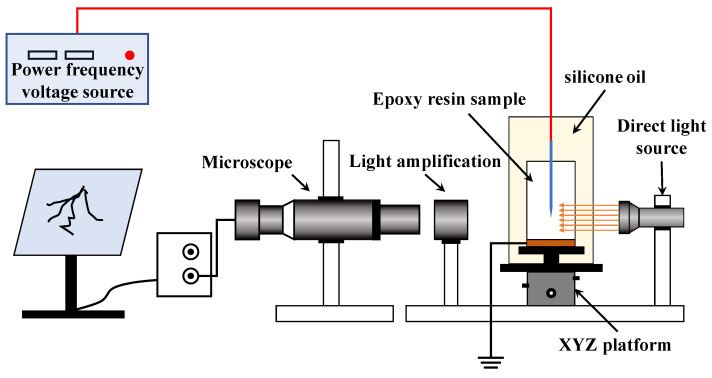
Electrical tree online observation system.

The real-time observing system of the electrical tree is shown in Figure 3. The alcohol-wiped specimens were placed in a clear glass container, using dimethyl silicone oil to fix them, and to ensure that the silicone oil was not over the specimen and the wiring port. The role of silicone oil was to prevent the specimens from flashing along the surface during the voltage addition process, while ensuring the visibility of the observation system. The temperature controller was placed in the vessel, and the temperature was adjusted to within the normal range (room temperature 20~25 °C). In order to facilitate adjustment of the specimen position, the glass vessel was fixed on a movable XYZ platform. During the experiment, an I.F. voltage was applied through the needle electrode, while the ground electrode was effectively grounded. The microscope observation lens was placed horizontally and connected to the monitor through the CCD camera to ensure that the observation-imaging path was in a horizontal straight line. The observation system was able to photograph and record the entire electrical tree state at the tip of the needle electrode [19,20].

Electrical tree test experimental method.

(1) Electrical tree initiation test: An AC voltage was applied to the epoxy resin specimen through the “needle-plate” electrode model. Beginning from 6 kV, the voltage was increased by 1 kV every 30 s until an electrical tree was triggered at the tip of the needle; then, the AC power was immediately turned off. The value at the moment of initiation was recorded as the specimen electrical tree starting voltage.
(2) Electrical tree growth test: After an electrical tree initiated, each specimen received an 18-kilovolt AC frequency voltage. At the same time, the growth of the electrical tree was recorded using a microscopic imaging system at a frequency of 10 s/frame.

In order to reduce the randomness of the test process and increase the reliability of the experimental results, 15 sets of tests were conducted for each ratio.

## 3. Results

### 3.1. Electrical Tree Initiating Characteristics

**Figure 4:**
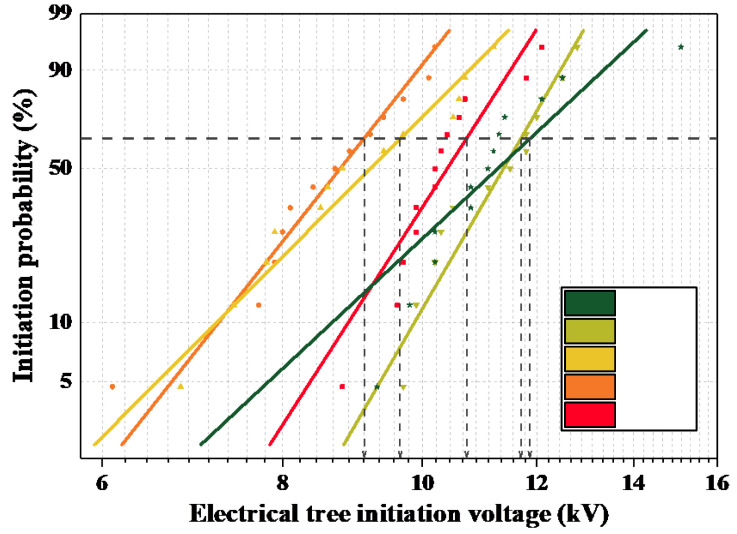
Weibull distribution of electrical tree initiation voltages.

In order to reduce the bias of the results caused by the boosting voltage rate, a 1 kv/30 s uniform boosting voltage rate was used in this paper. Whenever an electrical tree of 10 μm in length was observed, it was effectively considered to be initiated at that time. Weibull analysis was performed on the initiation voltage, and the initiation voltage that corresponded to 63.2% was taken as the characteristic initiation voltage of the specimen. The results of the Weibull analysis of the initiation voltages of the electrical trees in each epoxy system at room temperature are shown in Figure 4.

The electrical tree initiation voltages obtained from the Weibull analysis are listed in Table 1. It can be seen that after the introduction of 10% equivalent MPA, the electrical tree initiation voltage of the epoxy system was lower than that of the petroleum-based control. However, with a further increase in MPA content, the electrical tree initiation voltage also increased gradually. Compared with the petroleum-based control group, the starting voltage improved when the MPA content was 30%; when the MPA content was 40%, the electrical tree initiation voltage was effectively increased by 11.11%. This indicates that the introduction of an appropriate amount of MPA can effectively improve the insulation performance of the epoxy system.

Since at the maximum electric field strength, the epoxy resin at the tip of the needle deteriorated to form an electrical tree, Emax was also approximated as its electrical tree initiating field strength. According to the Mason formula, Emax was calculated as shown in Equation (1):

Emax = (2U_P_)/[r ln (1 + 4d/r)] (1)

U_P_ is the peak value of the applied voltage when the electrical tree is triggered, i.e., U_P_ = √2U_i_; r is the radius of curvature of the needle tip; d is the needle-plate electrode spacing.

### 3.2. Electrical Tree Developing Characteristics

**Figure 5:**
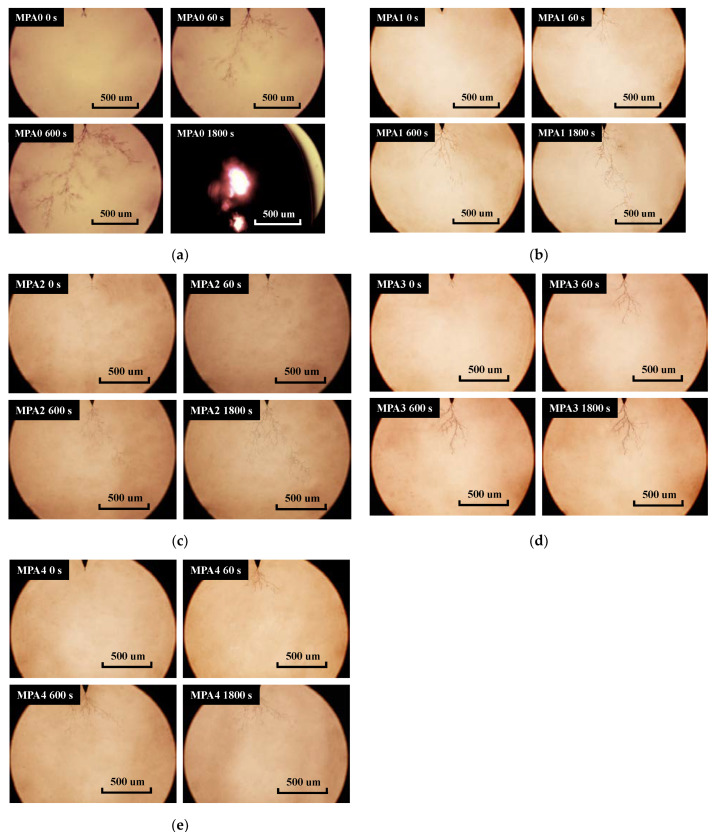
Propagation of each ratio of the electrical tree at 18 kV. (**a**) MPA0, (**b**) MPA1, (**c**) MPA2, (**d**) MPA3, (**e**) MPA4.

The growths of the electrical trees for different blending ratio epoxy resin systems under the power frequency voltage are shown in Figure 5. In this paper, the growth patterns at 0 s, 60 s, 600 s, and 1800 s after electrical tree initiation were taken for comparative studies. Under power frequency AC voltage, electrical trees generally developed rapidly along one or two channels at the time of initiation. With an increase in the voltage application time, new tiny branches grew around the main electrical tree, and new micro-branches were generated around the tiny branches as a result of the discharge phenomenon. Some of the tiny branches developed into trunk channels because of faster growth. The mesh-like electrical tree structure formed at the needle electrode developed continuously toward the flat electrode.

Comparing the electrical tree growth of each epoxy system, it can be seen that the electrical tree of MPA0 initially grew into a slender dendrite after initiation, and then the electrical tree developed in the direction of the electric field. The polymer molecules were continuously cleaved and gasified and accumulated heat under the action of a strong local field. Under high temperature and high voltage, the trunk channels of the electrical tree thickened and deepened in color, and many burr-like branches appeared on the trunk, forming a typical “pine branch” structure. Under 18-kilovolt AC voltage, 8 out of 15 MPA0 electrical tree specimens broke down at the side, or formed through channels between the electrodes of the needle plate. After the introduction of MPA, the electrical trees showed a dendritic structure, and the channels became lighter in color and narrower in width. As shown in Figure 5e, the trunk of the electrical tree with MPA 4 was shorter, with thinner branches that were more densely distributed. The experiments show that the growth direction of electrical trees is random, and does not strictly follow a direction that is perpendicular to the flat electrode.

**Figure 6:**
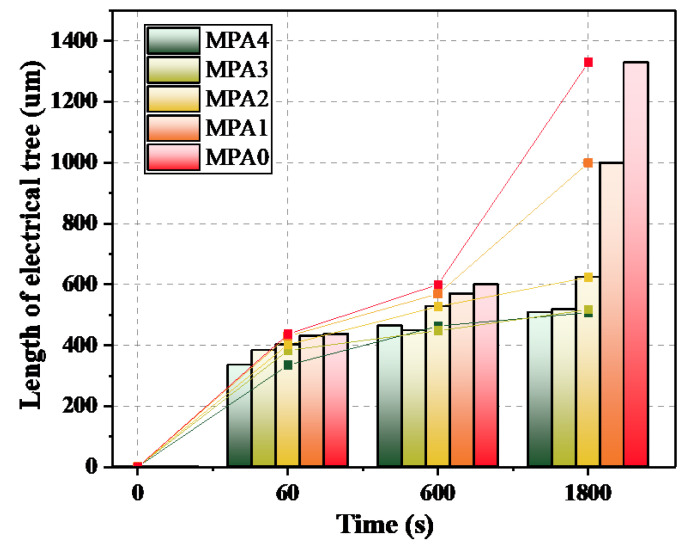
Relationship between the growth lengths of electrical trees and time for each MPA ratio.

The growth of each group of electrical tree specimens was counted and averaged, and the growth length of the electrical tree as a function of time was plotted, as shown in Figure 6. When the voltage applied time was less than 600 s, all five groups of specimens grew rapidly in a dendritic manner. However, with an increase in MPA content, the growth rate of the electrical tree in the epoxy system tended to become slower. At 60 s, the growth length of MPA4 was 76.7% of MPA0; at 600 s, the growth length of MPA4 was 77.2% of MPA0. With the application of voltage over a longer time, the MPA0 and MPA1 groups went into an accelerated growth state, while the MPA2, MPA3, and MPA4 groups all went into a stagnant growth state, with a growth rate that was close to zero. During the stagnant growth phase, the total length of the electrical tree was basically unchanged, but the channel width became slightly widened, and the trace of electrical tree cracking gradually deepened. After 1800 s, the electrical tree of MPA4 grew to 507.20 μm, while at that time the petroleum-based control grew to 1329.57 μm, and even showed breakdown between the needle-plate electrodes. Therefore, the introduction of MPA not only effectively controlled the growth rate of electrical trees, but also significantly inhibited the growth of electrical trees.

## 4. Discussion

### 4.1. Microstructure of Blended Systems

**Figure 7:**
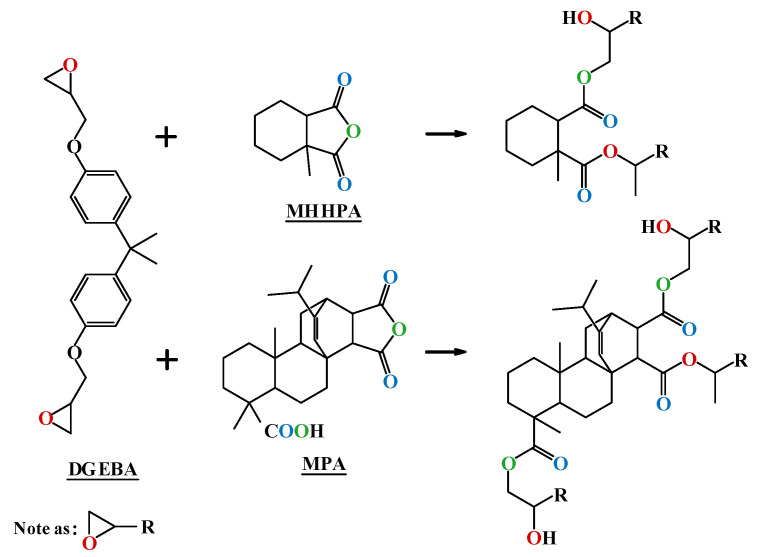
Molecular structures and cross-linking of DGEBA, MPA, and MMHPA.

In order to investigate the relationship between the macroscopic properties and the microscopic molecular structure of the epoxy system, molecular dynamics (MD) simulations were performed for each epoxy system to analyze the free volume ratio and the system energy change. The molecular structure and cross-linking principle of epoxy resins are shown in Figure 7.

**Figure 8:**
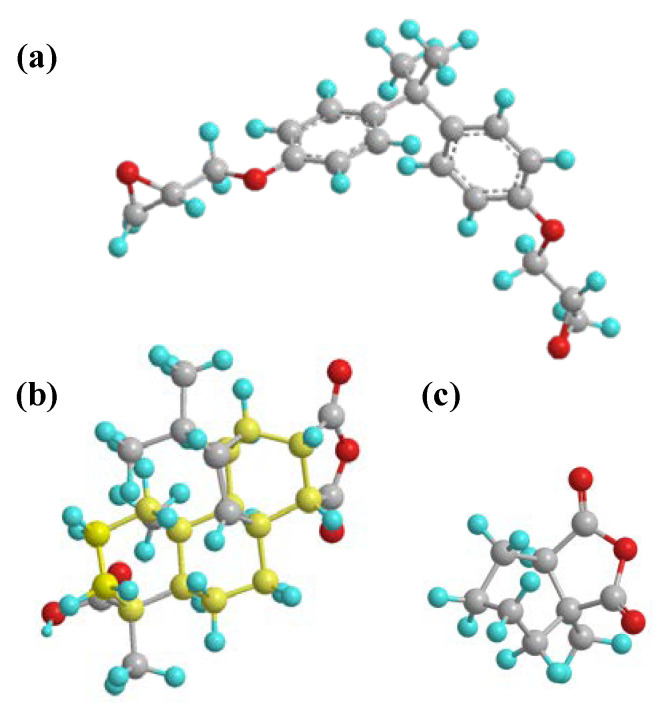
Three-dimensional structures of DGEBA, MPA, and MHHPA. (**a**) DGEBA, (**b**) MPA, (**c**) MHHPA. In the figure, the gray spheres represent C atoms; the yellow spheres represent the C atoms that make up the phenanthrene ring; the blue spheres represent H atoms; and the red spheres represent O atoms.

The monomer molecules of DGEBA, MPA, and MHHPA were constructed using Materials Studio (MS) molecular simulation software, and the geometric configuration was optimized for each of the three molecules by the Dmol3 module, as shown in Figure 8. Finally, the Amorphous Cell module was used to construct three-dimensional periodic amorphous cell models for each of the five blending ratios according to the content ratio of each component shown in Table 2. The density of each of these cell models is 0.6 g/cm^3^.

Using the Forcite module, the dynamics of the constructed material model were optimized: first, the NVT system synthesis was selected for cyclic annealing treatment from 300 K to 1200 K; then the model was minimized for energy and the molecular model with the lowest energy was selected; constant temperature and pressure (NPT) molecular dynamics treatment was used to eliminate the internal stress of the molecular structure. The simulated temperature was 298 K, and the simulated force field used was the COMPASS II force field [20,21].

**Figure 9:**
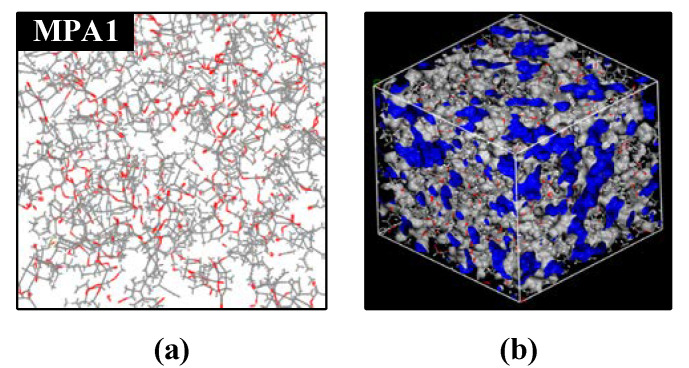
Molecular cross-linking mode and free volume ratio model of MPA1. (**a**) Molecular cross-linking mode, (**b**) free volume ratio model.

Finally, the cross-coupling simulation of the cells was scripted in Peal language. The solidified coupling model with 90% or more cross-linking is shown in Figure 9a.

**Figure 10:**
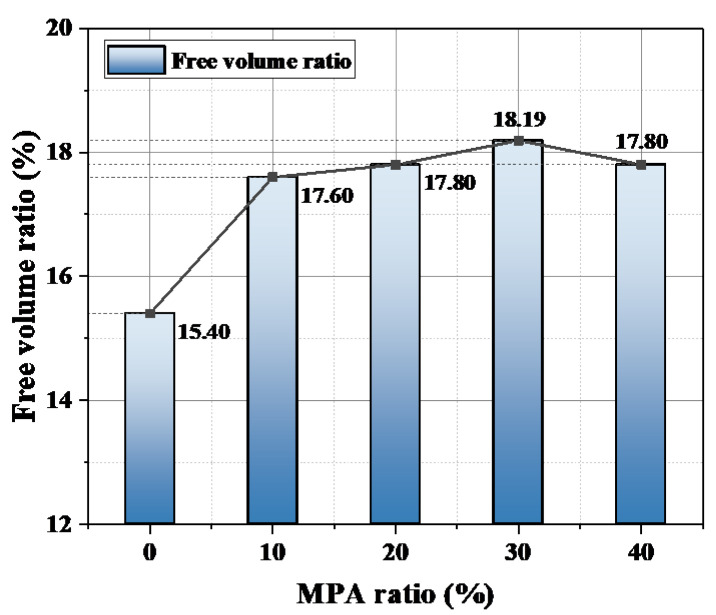
Effect of MPA content on the free volume of the blended system.

The Atom Volume & Surface tool was used to create the Connolly Surface, as shown in Figure 9b, and the free volume share of the solidified model was calculated according to Equation (2):

γ = V_f_/(V_0_ + V_f_) (2)

where γ is the free volume share; V_f_ is the volume in which the molecular chain can move freely; and V_0_ is the volume occupied by the molecular chain [22]. The effect of MPA content on the free volume of the epoxy system is shown in Figure 10. When MPA was introduced, the free volume of the epoxy system increased, and the free volume share of MPA1 increased by 2.20% compared with that of the petroleum-based control. Once MPA was introduced, the effect on the free volume was not obvious when the MPA content was between 10–40%, and the free volume percentage of the blended system tended to be stable.

**Figure 11:**
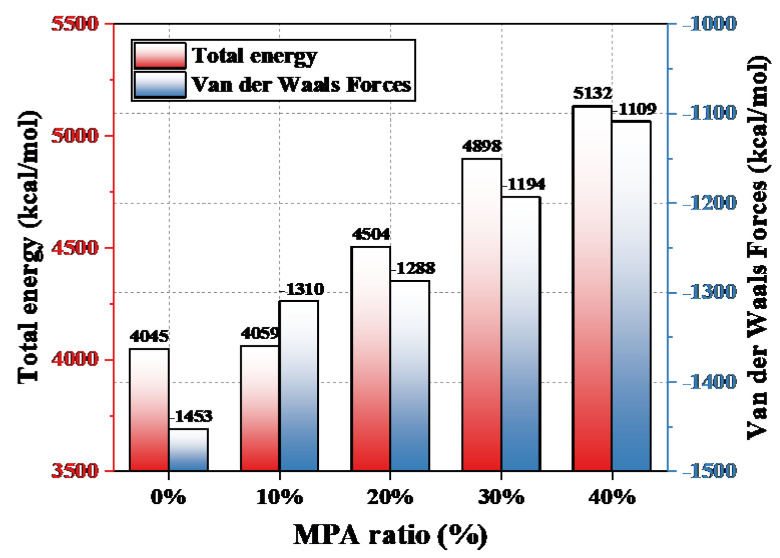
Effects of MPA content on the total energy and van der Waals force.

Based on the COMPASS force field, the variation in the total energy and van der Waals force of the epoxy system with MPA content were calculated, as shown in Figure 11. With an increase in MPA content, the total energy and van der Waals force of the epoxy system both showed an increasing trend. The total energy of the epoxy system is the sum of the bond energy and bond angle energy of the molecule. As can be seen from the figure, compared with the petroleum-based control group, when the MPA content was 40%, the total sum of bond energy and bond angle energy increased by 26.88%, and the van der Waals force increased by 23.68%.

### 4.2. Discussion of Electrical Tree Initiation Characteristics

Electrical treeing is a pre-breakdown discharge phenomenon. Under the applied AC field strength, the periodic injection and withdrawal of electrons causes localized field concentrations at internal defects such as at impurities and micropores in the insulation material, and this triggers local discharge [19]. The impact of the charged particles that are generated by the partial discharge makes the polymer chain of the insulating material break and rapidly oxidize, decomposing into small molecules [15,23]. Under the cumulative effect of the partial discharge, a slender electrical corrosion channel is formed in the insulating medium, i.e., an electrical tree.

Under power frequency AC voltage, electrons and holes are injected into the epoxy resin specimen in the form of field-induced thermal electron emissions away from the needle electrode through the Schottky and tunneling effects. In the early stages of electrical tree development, the thermal and chemical corrosion that are caused by partial discharge have an important impact on the insulation environment in the needle tip region. According to Figure 4, it can be seen that with an increase in MPA content, the initiation voltage of the electrical tree shows a trend of initially decreasing and then increasing. From the free volume results of molecular simulation, it is known that the free volume percentage of the polymer is higher than that of the petroleum-based control group after the introduction of MPA, as shown in Figure 9. In the free volume of the medium, the carriers that are injected and withdrawn are accelerated to form “hot electrons”, which continuously “hit” the polymer chains and “melt” the insulating medium locally. Therefore, when the free volume ratio is large, the average free travel of the partially injected electrons in the low-density region of the polymer is longer; furthermore, more energy is obtained from the electric field, which has a greater impact on the polymer and makes it easier for the partial discharge phenomenon to occur. Therefore, when MPA is introduced, the starting tree voltage initially shows a decreasing trend with a decrease in the free volume ratio.

As a result of the free volume percentage of the blended system changing slightly with a further increase in MPA content, the free volume percentage is not the main factor that affects the initiation voltage of the blended system when the MPA content is large. The gradual decrease in the initiation voltage is mainly caused by the gradually increasing amount of hydrogen phenanthrene rings. The hydrogen phenanthrene ring structure, as a strong polar molecular side group, increases the intermolecular chain forces as well as the rigidity of the polymer. According to Maxwell’s electro-mechanical stress theory, the structure of the hydrogen phenanthrene rings inhibits change in the molecular chain arrangement structure, which is not conducive to the development of electrical tree discharge channels. Therefore, the tree-initiating voltage of the epoxy system increases with an increase in MPA content.

In summary, when the MPA content is ≤10%, the free volume percentage in the epoxy system plays a major role in the initiation voltage; in other words, as the MPA content increases, the free volume percentage increases, the more easily the electrical tree is triggered, and the initiation voltage becomes lower. When the MPA content is ≥20%, the polar structure of the hydrogen phenanthrene ring increases the molecular rigidity, and thus inhibits the generation of electrical trees; in other words, as the MPA content increases, the hydrogen phenanthrene ring content, the stress threshold of the epoxy system, the dielectric cracking difficulty, and the tree initiation voltage all increase.

### 4.3. Discussion of Electrical Tree Growth Characteristics

As an electrical tree grows, new strong field areas are formed at the tips of the branches, which can be equated to new needle tips. Under the action of the alternating electric field, the continuously injected and extracted charges bombard the composite material in the form of “high-energy electrons”, forming new fine channels in the insulating material, and making the electrical tree develop towards the “flat” electrode.

Under power frequency AC voltage, at the tip of the needle and the tip of the electrical tree, Maxwell stresses that are perpendicular to the direction of the electric field are induced. In the later stages of development of the electrical tree, the mechanical stress in the discharge channel becomes the main influence on the development of the electrical tree, or even on the breakdown of the insulation material [19,23]. The Maxwell stress generated by the electric field accelerates the fatigue of the specimen, and when the stress exceeds the stress limit of the insulating material, small cracks are induced in the specimen. The presence of cracks causes mechanical damage and stress concentration in the specimen, which facilitates the acceleration of electrons and their impact on the polymer, thereby accelerating the breakage of chemical bonds and weak chains, leading to cracking of the polymer [23,24].

The molecular chain of the rosin-based curing agent MPA contains a large amount of polar hydrogen phenanthrene ring structures. The hydrogen phenanthrene ring (as shown in the yellow structure in Figure 8b) acts as an asymmetric polar side group that effectively increases the intermolecular forces of the epoxy system and, accordingly, enhances the cohesive energy density of the epoxy system [25]. As shown in Figure 11, according to the molecular simulation results, it can be seen that the introduction of MPA effectively increases the bond energy, bond angle energy, and the intermolecular van der Waals forces of molecules; as a result, the occurrence of Maxwell stress-induced dielectric cracking gradually becomes more difficult. Therefore, when the applied voltage time exceeded 60 s, MPA significantly inhibited the development rate and growth length of electrical trees in the epoxy-blended system, as shown in Figure 6.

## 5. Conclusions

In this paper, the electrical tree initiation and growth characteristics of a bisphenol A epoxy resin/maleopimaric anhydride curing system at power frequency AC voltage were investigated. The relevant experimental findings are as follows:

1. With an increase in MPA content, the free volume percentage of the curing system showed a trend of initially increasing and then stabilizing. MPA introduces a large number of hydrogen phenanthrene ring polar side groups to the blended system; as a result, the bond energy and bond angle energy of the blended system gradually increase, and the van der Waals force also increases simultaneously;
2. With an increase in MPA content, the initiation voltage of the electrical tree showed a trend of initially decreasing and then increasing; the initiation voltage of MPA4 increased by 11.11% compared with that of the petroleum-based control group. With an increase in MPA content, when MPA ≤ 10%, the initiation voltage decreased due to a decrease in the free volume ratio of the blended system; when MPA ≥ 20%, the initiation voltage continued to increase as a result of an increase in molecular rigidity.
3. The rigid structure of the hydrogen phenanthrene ring in MPA has an inhibitory effect on the growth of electrical trees. The electrical trees of the petroleum-based control group showed “branch-and-pine” shapes, with faster development and darker color under 18-kilovolt AC voltage. After the introduction of MPA, the electrical trees showed “branch” shapes with cross-colors and narrow widths. When the MPA content exceeded 20%, the hysteresis phenomenon appeared after 600 s.

## Figures and Tables

**Table 1:** Initiation voltages and initiation field strengths of electrical trees from different co-mixing ratios.

Sample	MPA0	MPA1	MPA2	MPA3	MPA4
Ui ^1^/kV	10.80	9.26	9.82	11.78	12.04
Emax ^2^/kV·mm^−1^	1291.06	1107.02	1173.94	1408.66	1439.37

^1^ Ui is the effective value of the electrical tree initiation voltage. ^2^ Emax is the maximum electric field strength of the needle tip.

**Table 2:** The mass ratio of each component at different blending ratios.

Reagents	MPA0 ^1^ (K = 0%)	MPA1 (K = 10%)	MPA2 (K = 20%)	MPA3 (K = 30%)	MPA4 (K = 40%)
DGEBA	100	100	100	100	100
MPA	0	7.58	15.82	24.80	34.62
MHHPA	72.85	68.27	63.29	57.86	51.93
DMP-30	0.86	0.87	0.89	0.91	0.93

^1^ Epoxy system in which the amount of anhydride provided by MPA is 0% of the total amount of anhydride in all curing agents.

## Data Availability

The data presented in this study are available on request from the first authors and from corresponding authors.
